# Minireview on the Connections between the Neuropsychiatric and Dental Disorders: Current Perspectives and the Possible Relevance of Oxidative Stress and Other Factors

**DOI:** 10.1155/2020/6702314

**Published:** 2020-06-30

**Authors:** Alin Ciobica, Manuela Padurariu, Alexandrina Curpan, Iulia Antioch, Roxana Chirita, Cristinel Stefanescu, Alina-Costina Luca, Mihoko Tomida

**Affiliations:** ^1^Department of Research, Faculty of Biology, “Alexandru Ioan Cuza” University of Iasi, Carol I Avenue, 20A, Iasi, Romania; ^2^Faculty of Medicine, “Gr. T. Popa” University of Medicine and Pharmacy, 16th University Street, Iasi, Romania; ^3^Department of Biology, Faculty of Biology, “Alexandru Ioan Cuza” University of Iași, Bd. Carol I, 20A, 700505 Iași, Romania; ^4^Department of Oral Science, Matsumoto Dental University, Shiojiri, Japan

## Abstract

Although the connections between neuropsychiatric and dental disorders attracted the attention of some research groups for more than 50 years now, there is a general opinion in the literature that it remains a clearly understudied and underrated topic, with many unknowns and a multitude of challenges for the specialists working in both these areas of research. In this way, considering the previous experience of our groups in these individual matters which are combined here, we are summarizing in this minireport the current status of knowledge on the connections between neuropsychiatric and dental manifestations, as well as some general ideas on how oxidative stress, pain, music therapy or even irritable bowel syndrome-related manifestations could be relevant in this current context and summarize some current approaches in this matter.

## 1. Introduction on the Neuropsychiatric—Stomatological Interactions

Dental disorders have a multifactorial origin, being correlated with an umbrella of risk factors and disorders going from the existence of transmitters in the teeth, oral infestation by insects or worms [1], feeling that the upper part of the mouth is pushing to the brains (press case report by our group) to the faulty personal hygiene in dementia [2], schizophrenia [3] or mental retardation [4], and peaking with the removal of all healthy teeth, e.g., in schizophrenia, in just 6 months and refusal of prosthodontic treatment [5]. Risk factors for a bereft dental hygiene even in healthy patients with an otherwise healthy oral state are vitamin B deficits related to alcohol abuse [6], considering that alcohol and drug consumption is toxic per se for teeth, and perhaps oxidative stress implications in this context [7, 8], as well as the lack of an appropriate and equilibrated diet.

Classical risk factors for teeth decay and periodontal problems are represented by various body dysmorphic disorders [9] and psychosomatic delusions (e.g., halitophobia or even phantom bite syndrome [1, 10, 11] together with cenesthopathies (e.g., various types of abnormal sensations without somatic aberrant findings—some of these complaints being listed in Umezaki and his team's article and classified according to the Diagnostic and Statistical Manual of Mental Disorders—5 (DMS-5) criteria as delusional disorders, somatic type (DDST) [12] as well as anxiety and panic-attacks-associated dryness (xerostomia) of the oral mucosa and reduced saliva [13, 14]. A specific example is the paper of Takenoshita's group from 2010, which clearly demonstrated that from 162 patients presenting burning mouth syndrome (BMS) and atypical odontalgia (AO) (both somatoform disorders referring to pain without a clear organic cause) most of them were likely to exhibit mood or affective disorders in the AO case versus neurotic and stress-related manifestations—more common in BMS patients [15]. Also, the influence of dental lead and cadmium (and heavy metals in general) on the neuropsychiatric manifestations were illustrated in a six-year-old patient [16]. In the case of some neuropsychiatric disorders, impaired social and/or financial deficits as a consequence of the disorder causes a reduced access to dental care or in the case of autism spectrum disorders, which by the nature of the disorder makes difficult for the dental specialist to even get close to the mouth of the patient.

The most common neuropsychiatric disorder that has an impact on the oral health is anxiety with almost 50% of patients known to exhibit different levels of anxiety on their dental visits [15] measured by dental anxiety scales developed to evaluate such manifestations [17, 18] and with aesthetic matters and affected self-image (e.g., dentition aspect) contributing to the levels of anxiety and depression-like behaviors [1]. In addition, some studies suggest that the specific dental anxiety is not even a component of their classical general anxiety disorders family but rather belongs to the group of mood disorders by using specific scales such as dental anxiety scale and Patient Health Questionnaire. Therefore, the authors accentuate/emphasize the fact that this might represent a very specific manifestation and not just a simple diversification of other phobias, such as those for blood and injections [19].

**Figure 1:**
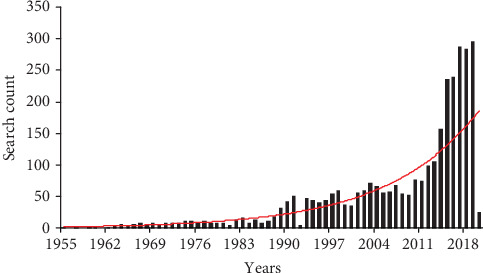
The timeline from 1955 to 2020 of the search count by using the keywords dental and psychiatry. There has been observed a significant increase of interest in these fields illustrated by the trendline with the highest search count till now recorded in 2019-297 entries.

While the interest in the interactions that might appear between neuropsychiatric and dental disorders is not exactly new, with specific scales for dental anxiety being designed to study these aspects in the ‘70s [17, 18], there is a general consensus in the literature that the connections between these two aspects are largely unknown, unrecognized, underacknowledged, and most importantly insufficiently tackled by the practitioners in both these medical areas [13–15]. The interest regarding the interactions that might appear between these two fields is described in Figure 1 under the form of a timeline from 1955 to 2020 of the search count by using the keywords dental and psychiatry. There has been observed a significant increase of interest in these fields illustrated by the trendline with the highest search count till now recorded in 2019-297 entries.

Therefore, the literature contains some scarce original research studies [15, 19] some case presentations [16, 20] as well as some reviewing [13, 14] on the current data, which are stating clearly the necessity for further efforts in understanding the correlations between neuropsychiatric and dental manifestations, as well as some attempts in establishing a few preliminary guidelines in this matter [21, 22].

One approach encompasses how some HIV-related neuropsychiatric manifestations could affect the dental practice supported by some case presentations [23], as well as specific approaches on “dental neuroplasticity, neuro-pulpal interactions, and nerve regeneration” [24].

Two case presentations by Kenrad group in Denmark describe some original approached of possible correlations between morphology and eruption location of permanent maxillary incisors along with other cranial bone malformations and some neuropsychiatric manifestations, such as attention deficit hyperactivity disorder (ADHD), deficit in attention, motor control and perception (DAMP), and mental retardation [20, 25], with the latter also exhibiting an increased rate of malformations in the oral area (e.g., palatoschisis, cheiloschisis, cheilo-gnato-palatoschisis) [26].

Some stomatological deficiencies related to bulimia and anorexia (which might be observed initially by the dental specialist) [1] might conclude with some serious damage of the teeth [13, 15].

Also, in a meta-analysis and systematic review conducted by Kisely and his team, the emphasis is put on the gravity of dental disease in people suffering from severe mental disorders [27]. As a result, they observed a 3.4 higher probability in the psychiatric population to be edentulous (absence of all teeth) compared to the general population. Also, the number of decayed, missing, filled teeth (DMFT) index quantifying lifetime dental caries experience was significantly higher in patients diagnosed with severe mental illnesses (mean difference 6.2, 95% confidence interval 0.6-11.8), when compared to nonpsychiatric patients. The follow-up meta-analysis and systematic review conducted later on in 2015 illustrated that the trend of a poor oral situation in psychiatric patients remained unchanged. Even if the odds for people suffering from severe mental disorders are lower than in the previous findings, they are still 2.8 times higher than in the general population [28]. Despite this apparent improvement in DMFT values and the index following dental surfaces, decay, missing, or filled surfaces (DMFS index), the general tendency is to keep the same significantly increased values with DMFS maintaining the same mean differences rates [28].

The group led by Kisely more recently focused on specific psychiatric illnesses, such as depression, anxiety, and dental phobia, and their findings are similar to their previous ones—increased both DMFT and DMFS indexes together with increased tooth loss as opposed to the general population. With no other association being mentioned, the periodontal disease seems to be connected only with panic disorder [14].

Oral health represents a concern in other countries as well with Zusman and his team reporting an alarming percentage of 69% of patients presenting only partial natural dentition compared to the total amount of people institutionalized in psychiatric care and 29% being edentulous [29] in Israel and in Denmark, where two-thirds of mature people diagnosed with mental illnesses were reported to be edentulous [30].

Taking into account, many of the neuropsychiatric conditions are described in relation with a genetic defect of some sort (for instance, the implications of gene TAOK2 in autism spectrum disorders, schizophrenia, and other neurodevelopmental affections [31]); a new approach in studying neuronal differentiation occurred. Goorha and Reiter proposed an innovative war to harvest cells for neuronal culture by using the dental pulp of deciduous teeth in order to develop neurons for *in vitro* studying of neuronal populations reactivity—a critical perspective required to investigate cellular and gene expression modifications that appear in neuropsychiatric pathology [32].

All the studies mentioned above highlight the necessity of instating collaboration between the psychiatrist and dental professional. Some of these teams also tried to come up with solutions for the stomatological problems encountered in psychiatric patients, for example, Zusman and his team propose in hospital dental treatment (by pointing out that is a cost-efficient method and would also encourage preventive treatment) as dentist prefer extraction due to the collaboration difficulty with patients suffering from mental disorders. For this purpose, there has been developed an assessment tool whose usage potential was analyzed by the Oral Health Assessment Tool which proved to be valid and reliable in screening persons committed to care facilities and can be applied even in people suffering from cognitive impairments [33].

## 2. Mechanistical Aspects

For the purpose of this paper, studies were searched mainly in the Pubmed database by using the keywords neuropsychiatric, dental disorders, oxidative stress, pain, music therapy, and irritable bowel syndrome with cross-references of these keywords also counted in. By conducting our search, we observed that the number of searches using the keywords dental and psychiatry significantly increased over the years following 1955-2020 timeline, the highest search count being registered in 2019-297, with this being portrayed in Figure 1 together with the trendline designed to better illustrate the increasing interest in the connection between these medical fields.

By comprising a series of articles (research papers, reviews, case reports, books), we obtained Table 1 which illustrates in a clearer demeanor the possible interactions between certain psychiatric disorders (such as depression, anxiety, schizophrenia) and various dental manifestations highlighting the risk factors as the possible bridge that connects the two different fields which have more in common than just the close localizations of the two anatomical areas involved.

Therefore, one of the possible causes for all the aspects mentioned in the table could also be represented also by the treatment administrated for most of the neuropsychiatric disorders, as some of these medications can interact with drugs used in dentistry [1] and also since it was showed before for example, that specific drugs such as lithium [34] could generate xerostomia (dry month, as previously mentioned) and stomatitis, while this could represent a general effect for most of the neuropsychiatric treatments such as antipsychotics, antidepressants (e.g., selective serotonin reuptake inhibitors: SSRIs) or mood stabilizers [14].

In fact, there is a review work in this specific matter (the effects of neurological medication on salivary secretion and further on dental care), by a Romanian group [52], which extensively revised some of the 400 available drugs in this area known to affect salivary secretion, that were separated by authors in “strong” and “insignificant”, as based on their effect on salivary secretion [52].

The reason behind dental phobia might be attributed to fear of experiencing pain. Considering that severe psychiatric illnesses are always under a specific treatment course, medication interactions between dental and psychiatric treatment might result in a diminution of anesthesia effect (such as in the case of patients medicated with SSRIs medication, with fluoxetine, paroxetine presenting the highest influence when combined with codeine or derivatives) [53] which results in pain perceived during the treatment provided by the dental practitioner.

Mechanistically, it is believed that decreased salivary secretion/xerostomia could be incriminated for increased calculus and plaque formation, as well as for significantly higher levels of dental decay and periodontitis [13]. On the other side, clozapine, an atypical antipsychotic, was cited for example for inducing the opposite sialorrhea (exaggerated production of saliva) (as reviewed by Bharti et al. [13]).

The difficulties to treat psychiatric patients could also be relevant for the phobic states, considering that oftentimes cancellation of most of their appointments, sometimes in the last moment is frequent [1], in their case being indicated to apply some of the approaches and current knowledge in this matter that will be described in the last chapter.

Also, mechanically and psychologically speaking, as well as in the case of dental anxiety, the process is mainly relying on several classical psychological fundaments, such as the fear of unknown, previous unpleasant experiences with dental interventions and a personal increased vulnerability of anxiety manifestations [1].

Saliva, a biological fluid underexplored, has proven to be a really suitable alternative, noninvasive, nonpainful, and relatively easy to sample, to evaluate several markers. This biofluid shares 25-30% of proteins with blood, an aspect which clearly comes as an advantage [54], in regard to repeated samplings, accessibility, and precision of the determination [55]. As it is emphasized in Podzimek's paper, salivary markers are on the verge of becoming a diagnostic tool for periodontal disease but also for general diseases. This is not such a novelty as it was demonstrated even in 1978 that salivary concentration of prostaglandins, namely prostaglandin E2, prostaglandin D2, and prostaglandin F2*α*, are markers significantly increased in patients suffering from major depression [56].

A correlation between high levels of prostaglandins found in salivary products and severity of depressive conditions was highlighted as an intriguing perspective considering that central nervous system prostaglandins influence pain perception, behavioral cues, and other aspects such as sleep cycle and food intake [57].

Inflammation is correlated with oral pathology, such as periodontal disease (its progression is measured by pocket depth, gingival bleeding and suppuration) [58]. Salivary evaluations indicate towards an increased level of interleukin (IL)-1*β* during periodontitis, correlated with the progression of the disease and also indicating its active or inactive status. Also, IL-1*β* was linked to advanced periodontitis [59].

On the other hand, psychiatric pathologies such as depression have been linked to inflammation processes with incrementations of IL-1*β*, the same interleukin implicated in periodontal disease, as mentioned before, along with high levels of IL-6, tumor necrosis factor (TNF) and C-reactive protein (CRP) in peripheral blood assays [60]. Individuals suffering from periodontitis also present high levels of IL-6 as compared to healthy controls. Amongst other roles, IL-6 found in the salivary fluid presented itself with stimulating proprieties towards differentiation of osteoclast cell population and bone resorption, both processes associated with the progression of peri-implant disease [61].

Another inflammation marker evidenced in psychiatric patients, TNF, is also remarked its presence in generalized chronic periodontitis [62], indicating again towards an association between these two pathologies.

Noteworthy, is that blocking cytokines, like TNF, or signaling molecules part of inflammatory pathways, for instance cyclooxygenase 2, demonstrated to result in a decrease of depressive symptoms in patients with manifesting major depression, as well as other medical disorders among them, rheumatoid arthritis, psoriasis, and cancer [63, 64]. At the same time, CRP, a long-known inflammatory indicator presenting increased levels in periodontal disease (plasma as well as salivary levels), after anti-inflammatory treatment displays decreased levels, which is an indicator of a successful treatment [55]. Therefore, it is fairly possible that efficient treatment of periodontal disorders in psychiatric patients might not only benefit from an oral health improvement but also from an improvement of their psychiatric symptoms. All of these could be very important to be tackled in order to manage the correlations between neuropsychiatric and dental manifestations.

The management of dental disorders in the context of severe psychiatric diseases such as major depression was a matter of concern for decades ago. This is proven by a study published in 1991 following the management of dental problems manifested in people suffering from major depression and trying to pinpoint the factors that led to these issues [65].

Aside from the pharmacological and neurological aspects that link neuropsychiatric disorders to dental disorders, one key factor that needs to be taken into account is related to dental habits along with dental hygiene, frequency of visits to a dental clinic as well as socioeconomic status. One study in particular observed a loss of interest regarding the performance of hygiene activities, oral hygiene included, as well as an appetite and weight change in patients with moderate depression [50]. Another study conducted on 7305 adolescents, from Norway, chose to exclude subjects representing a minority group with both parents born outside Norway as it was previously observed that food consumption pattern and mental distress is different from natives. They concluded that there is a strong association between soft drink consumption and mental distress [48], whereas the case report on cola dependency in a woman with recurrent depression illustrated that overconsumption of caffeinated drinks might accentuate the psychiatric symptoms [49]. Psychiatric patients institutionalized for longer periods of time are entitled to at least 1 dental visit per year, as illustrated by Zusman in Israel population with the possibility of the patients to refuse the visit which might be driven by a certain level of anxiety and/or phobias [29], while in Denmark population, in a study of elderly psychiatric patients, it was illustrated that the difficulty to work with them puts them in a high risk for oral problems as they can refuse even daily dental care assistance [30].

In the case of eating disorders, compared to a healthy population, there is a higher risk of teeth erosion due to acidic fruits and drinks alongside frequent vomiting and gastric reflux leading to an increased number of decayed, missing, and filled surfaces [14].

As highlighted by Becker, there are numerous interactions that might occur during the dental treatment of a medicated psychiatric patient [53]. Therefore, there is a need of a closer collaboration between these two professionals. Nonetheless, the difference in pain perception acknowledged to be present in patients suffering from psychiatric disorders must not be overlooked [66].

## 3. The Possible Relevance of Oxidative Stress, Pain, Music Therapy and Irritable Bowel Syndrome-Related Manifestations in the Interactions between Neuropsychiatric and Dental Disorders

Thus, considering the aforementioned aspects and the previous experience of both our groups implicated in this minireview on the oxidative stress metabolism and its correlations with most of the neuropsychiatric disorders [67–74], but also modulations of oxidative stress status in some dental disorders [75–77] as well as our experience in studying pain processing in the neuropsychiatric context [66, 78, 79] and its possible correlations with music therapy [80], we decided to discuss at large in the present chapter the possible relevance of such parameters. Also adding up our gastrointestinal and brain-gut axis previous studies [81–85], we want to stress out the importance of oxidative stress status and its biomarkers, pain perception, music therapy and gastrointestinal and especially irritable bowel syndrome (IBS)-related manifestations and their interactions with neuropsychiatric and dental disorders.

### 3.1. Oxidative Stress

As mentioned in the previous section, inflammation has a role in both dental disorders and psychiatric manifestations and it is known to be accompanied by altered molecular processes, a prominent one being oxidative stress. Although reactive oxygen species occur in the physiological state as well, they become pathological when the equilibrium between oxidation and reduction processes is lost [86]. This fine balance is destroyed either by increments of reactive oxygen species, lack of antioxidant protection, or by both processes. The result of this imbalance in the case of periodontal disease is marked by significantly increased destruction of periodontal tissue [87]. For example, in regard to oxidative stress, we showed in the past that there is a similar antioxidant decrease (as expressed through superoxide dismutase and glutathione peroxidase specific activity from serum) in patients with both mild cognitive impairment (which is an intermediate state between normal aging and dementia) and Alzheimer's disease, correlated with a progressive increase of lipid peroxidation markers (such as malondialdehyde) from control patients to mild cognitive impaired ones and finally to the highest levels observed in Alzheimer's disease [67].

Similar progressive increase for the main oxidative stress markers in serum was observed, as based on the chronicity of the disease, also in the case of the major depressive disorder, with a progressive decrease of the main antioxidant enzymes such as superoxide dismutase and glutathione peroxidase in the recurrent depression group versus first episode group. Moreover, a significant increase in malondialdehyde concentration was observed in recurrent depression patients, as compared to the first episode group suggesting a progressive evolution of the oxidative stress metabolism modifications [69]. In the same way for the patients with temporomandibular joint disorders (TMD), our group showed a progressive importance of the oxidative stress status modifications from the synovial fluid and significant correlations between the severity of this disorder and some oxidative stress markers such as albumin [75]. With previous works in this area determining similar aspects for other oxidative markers such the superoxide radical [76], redox state of albumin [77] and even review works showing the modifications following this progressive context for a variety of markers, such as the classical inflammatory ones (e.g. cytokines), proteinase, arachidonic acid, neuropeptides, or nitric oxide [76], there is a clear connection between oxidative stress and TMD.

Thus, in this context, we can speculate here on the possible relevance of the oxidative stress status modifications and its complex implications in the aforementioned correlations between the neuropsychiatric manifestations and the dental deficiencies, especially considering that in 3 separate disorders presented above by our research groups, we could observe a progressive modification of some oxidative stress parameters, as related to the severity of mild cognitive impairment, Alzheimer's disease, major depressive disorder or the temporomandibular joint disorders [67, 69, 75]. Also, in this context, we could add here the paper of Gillberg et al. group [16] which showed, as described in the Introduction section, which it could be a correlation between some heavy metals (e.g., dental lead and cadmium) and some neuropsychiatric disorders children (case presentation).

However, regardless if there is or not a clear correlation between these aspects that could be mediated by the main oxidative stress markers modification, further studies following oxidative stress influence seem warranted.

### 3.2. Pain Processing

In regard to the pain processing, it is actually suggested by some authors that the nociceptive processing in the context of the correlations between the neuropsychiatric manifestations and the dental disorders could explain some of the controversial results we presented through this review. For instance, in the work of Takenoshita et al. where it was demonstrated, as mentioned, that a significative proportion of patients with some somatisations in this interaction context (e.g., BMS and AO) had no clear and definitely psychiatric diagnosis, although there were sent from psychiatric units with a possible diagnosis [15].

In this way, the aforementioned authors are mainly suggesting that pain could explain these differences, as for example, psychiatrist observed pain as a part of the general condition, while dental specialists are interested more in the location of the nociceptive process [15].

Also increased levels of anxiety are present quite often in patients with temporomandibular joint dysfunctions [1]. Even more, in this context, we could mention that our group previously demonstrated important correlations between nociceptive processing and oxidative stress manifestations, for example, through the mediation of the brain renin-angiotensin system [78, 79].

### 3.3. Music Therapy

Same goes regarding the music therapy usage in this context, as our group has previous experience in studies involving applied music therapy on the dental pain (same interactions mentioned above) [80]. The thresholds of pain on gingiva or buccal mucosa in the oral cavity were significantly higher when the subjects were listening to ballads or classical music than those without music. In the functional magnetic resonance imaging (fMRI) study, blood-oxygen-level-dependent (BOLD) deciduous signals in the cingulate cortex induced by pain were attenuated by listening to popular music, ballads, or classical music. Listening to music might reduce the pain stress [80]. Parasympathetic nervous activity values significantly increased under listening to ballads or classical music compared without sound. Sympathetic nervous activity values significantly decreased under listening to classical music compared without sound. When subjects heard classical music, there is an equilateral correlation between the pain threshold and the change of the parasympathetic nerve activity, and there is a negative correlation between the pain threshold and the change of the sympathetic nerve activity [88].

On the other side, in the neuroscience field, there is also an abrupt increased awareness regarding music therapy, with 2 papers for examples being published on this matter in Lancet [89] and Nature [90] only in the last years, as well as specific studies in this matter in our country with Miu group showing, for example, the influence of music in empathy, visual imagery, mood, general psychophysiological, or other affective approaches [91–94]. Even more, the aforementioned interactions could be observed in this case also, with papers demonstrating a clear correlation between music therapy and dental anxiety [95], as well as between dental pretreatment anxiety, stress levels, and dental hygiene [96].

Music therapy has a positive effect in the control of dental anxiety. Salivary cortisol, blood pressure, heart rate, and body temperature during dental treatment are significantly decreased with calming music compared with the treatment without music [95]. Waiting for a medical treatment can induce anxiety and may lead to the experience of stress. State anxiety levels with relaxing music decreased significantly compared to the state without music [96].

The activity of the muscle by the clenching causing temporomandibular disorder decreases when participants hear favorite music. To hear the favorite music or relaxing music decreases the level of the stress and increases the pleasure intensity [97]. Many cultures throughout history have relied on music to cure physiological or psychological ills. Music affects specific physiological processes such as cardiac output, respiratory rate, pulse rate, and blood pressure. Auditory stimuli may directly suppress pain neurologically and may mask the sound of the dental drill removing a source of conditioned anxiety, because music may induce relaxing effect. Therefore, the music is very effective in dental treatment [98].

Thus, further research seems warranted regarding the ways in which music therapy could modulate the aforementioned complicated interactions between the neuropsychiatric manifestations and the dental disorders mentioned above.

### 3.4. Gastrointestinal Implications—Irritable Bowel Syndrome

Regarding the gastrointestinal implications in this context (with a special focus on IBS—which is considered one of the most important and modern functional gastrointestinal syndromes), as it is diagnosed only based on symptomatologic criteria such as abdominal pain and changes in the pattern of bowel movements, in the lack of a clear underlying damage, despite the fact that IBS combined with a functional disorder, might lead to IBS symptoms becoming more severe and to an increased rate for psychopathologies [99].

There are some initial studies stating that these mechanistic connections that we are suggesting here could be feasible, as shown quite clearly in the Esmaillzadeh et al. [100] study regarding a correlation between tooth loss and irritable bowel syndrome, as well as other studies, such as the conclusive review of Kazemi et al. group [101] looking on the “oral health,” “masticatory performance,” “dental status,” and “eating” or “food intake” correlations or further work on the relevance microbiome of the oral mucosa in irritable bowel syndrome [102], but also on other gastro-intestinal manifestations such as the inflammatory bowel disease (e.g., Crohn's disease) in studies focusing mainly on the dental management of patients with inflammatory bowel disease [103], a disorder where oxidative stress and inflammation modification are much clearer, as we also showed previously [85].

We could mention here that lately there seems to be also a correlation between oxidative stress status (and even inflammation) and IBS, as stated by some recent publications in this area of research [104, 105]. Choghakhori and his team observed that in the case of IBS patients, the level of proinflammatory cytokines is increased (including TNF*α* and IL-17) and in contrast, the level of anti-inflammatory cytokines is decreased, modifications that were also present in the serum by a lower level of antioxidants and a higher level of malondialdehyde (MDA) [104]. On the other hand, the study conducted by Mete et al. illustrated a higher level of lipid peroxidation which might be due to an increased nitric oxide (NO) level and xanthine oxidase (XO) activity and a lower concentration of antioxidant enzymes [105].

Some studies have focused on the possible connection between IBS and TMD by pinpointing the correlation between facial pain and abdominal pain, such as the case-control study conducted by Nicola and his team [106] that portrayed a six times greater risk for TMD patients to develop IBS and in a more severe form compared to the healthy control, whereas the study of Gallotta et al. [107] illustrated a 3 times greater risk of IBS patients to develop TMD with the observation that patients who fulfilled both of TMD and IBS criteria seemed to also share a significant cooccurrence of psychiatric disorders.

## 4. Conclusions

Thus, as stressed out in this review, although it was clearly described that most of the neuro-psychiatric patients could have up to 2.7 higher risk of losing all their teeth [4], there is still a poor understanding of the connectivity between these two areas of research, as well as between the specialists working in these 2 fields.

Still, as mentioned by some authors [1], only quite recently there was an increased interest in introducing, for example, the study of some behavioral sciences in superior and normal dental training.

This could be very important, considering that the dental specialist could/should be among the first (if not the first), to observe and increase awareness in some cases such as those related to eating disorders (e.g., anorexia, bulimia), where an erosion of the dentition could be seen in more than 30% cases [13]. This is even more relevant in the context where these patients are generally unwilling to address a psychiatrist [14].

A better awareness about these connections and multidisciplinary approaches (e.g., teams composed by psychiatrists/neurologists/psychologists and/or dental specialists) in this matter are suggested [1, 13, 15], as they provide clear advantages for the patients facing the disorders described in this minireport.

In this context, lately, some guidelines for this matter (dental—neuropsychiatric interaction) emerged in the literature [22, 44], as well as some suggestions/approaches for the dental specialists referring for example to the risk of bleeding in Depakene-taking patients with epilepsy, awareness on poststroke anticoagulant medication, electric tooth-brush used in motor deficient Parkinson's disease patients, the associated temporo-mandibular dysfunctions which are observed also in Parkinson's disease pathology [22], the fact that the nitrous oxide can increase the risk of hallucinations in schizophrenic patients, how the dental specialist should cope with the memory deficits from dementia patients in dental care interventions or aspects regarding the low pain tolerance in substance-abuse patients [44].

There is a suggestion for campaigning some practical matters, such as an active implication in increasing number of specialists willing to treat patients with severe mental disorders [14]. There are also very modern [108], as well as classical [109] individual studies in the literature in this matter, suggesting, for example, the usage of some “multistep” regulations for dental care in children with neuropsychiatric disorders [108], as well as aspects on how to neutralize dental fear with neuro-linguistic programming [109], respectively.

Also, some authors are suggesting along with specific physical approach (e.g. the actual medication/drugs currently used in psychiatry), different psychological techniques/approaches, such as gradual and supportive psychotherapy, different types of behavioral therapy, including hypnosis [110, 111] or social therapy, rehabilitation, and community care [1].

Stress changes a body into an oxidation state, and the oxidation stress adversely affects the body such as the causes of the disease. Oral cavity induces some diseases such as temporomandibular disorder [75], trigeminal neuralgia, or tongue ache symptom.

On the other hand, the pain with infection or trauma becomes the severe stress. Therefore, it is important to find a therapy to relax stress and to relieve pain, which prevents disease. Thus, listening to music is a simple method to defuse stress or pain [80, 88], and it is effective in a mental disease patient, as mentioned above [89, 90].

Also, it is considered that dental specialists could actively explain to their patients the biological mechanisms implicated in the possible disorder, when referring them to a psychiatrist [15] while also having the knowledge for such specific psychiatric referral [1]. On the other side, they should refrain from considering chronic oral pain as probably “psychogenic” [15], being demonstrated that telling a patient nothing is wrong while they experience pain could result in very deficient disease management [1].

Even more, there are cases when although psychiatric disease is detected or suspected, it is quite hard to get to the next step of getting psychiatric support from a specialist [1]. In addition, we could mention in this context that some authors are drawing the attention on some incorrect diagnosis issues in this matter [20], as for example, in the case of the before-mentioned study of Takenoshita group, where it was found that 50.8% of BMS patients and 33.3% of AO patients had no specific diagnosis, although there were sent from psychiatric units, as being suspected for these observations [15]. It seems that these different observations could have pain as a central reason, as we explained in the previous chapter.

In addition, other studies are suggesting that perhaps some of these disorders, such as, for example, the specific dental anxiety, are not even a component of their classical general anxiety disorders family, but it rather belongs to the group of mood disorders [19].

It seems thus that there is an undoubtedly need for a better understanding on the interactions between these two areas of research, and a closer collaboration between specialists working in these two domains. There is definitely also a need for an individualized specific approach for these patients, with complex preventive and rehabilitation programs in this direction.

## Figures and Tables

**Table 1:** Different symptomatologies and their consequences/manifestations influenced by different risk factors with an accent on the correlation between various psychiatric and dental disorders, highlighting the possible comorbidity between these 2 areas based on several research articles, reviews, clinical trials, and case reports.

Manifestation/symptomatology	Risk factors	Consequences/correlations	Study type	Sample size	Specificity	Other observations
1 Depressive symptomatology	Not under treatment	Favorable environment for caries because of a decreased salivary flow	Double-bind trazodone + imipramine+ placebo, parallel group design [35]	379 patients (142 trazodone, 142 imipramine—positive control, 95 placebo) [35]	Tricyclic and heterocyclic antidepressants [35] Geriatric population [36]	The high incidence of certain side effects even in the placebo groups might have a connection with the neurotic symptomatology [35] Tricyclic and heterocyclic categories of antidepressants target anticholinergic activity by blocking parasympathetic salivary glands [35] The use of hyposalivatory medications increases with age [36] The anticholinergic side effects of classical tricyclic antidepressants are persistent [37]
	Undertreatment (tricyclic and heterocyclic categories)	Influences salivary flow [35–37]—creates xerostomia—> increased calculus and plaque formation, higher levels of dental decay and periodontitis [13]				
	High levels of prostaglandins (found in salivary products)	Atypical face pain, odontalgia, burning mouth syndrome, lupus erythematosus, general disorders of taste and salivation [1]				Affected hygiene and tobacco-associated usage Up to 30% more likely to lose all their teeth
	Severe depression acutizations	Atypical face pain and/or facial arthromyalgia [38], burning mouth syndrome [39, 40]	Two centre double blind clinical trial [41] Controlled trial	93 patients at the actual start of the patient [41] 50 patients (25 for BMS∗ group and 25 for control) [39]	No psychotic treatment for two weeks prior to the study [38] Chronic painful oral conditions [39]	Out of the 53 patients considered as “psychiatric cases” due to their symptomatology, only 17 were still classified as such at the end of the nine weeks study (51) 44% of the BMS∗ group presented an associated-psychiatric disorder compared to the control group (16%) (52)

2 Anxiety	Bruxism (tooth grinding)	TMJD∗, recurrent stomatitis or lichen planus [1]				
	Phobias	Increased presence of decayed teeth recorded by DMFT∗ and DMFS∗ indexes, increased tooth loss [14]				

3 Mildest dental irregularities	Psychological and psychiatric disturbances (anxiety manifestations)	Very disproportionate distress and depressive-social withdrawal, isolation and reduced self-esteem [1]				

4 Addiction on drugs and/or alcohol	Excessive bruxism (tooth grinding and toxic habits)	↑ Risk of oral cancers [13] Risk for caries [14, 42, 43]	Clinical trial [42]	28 subjects divided in 3 groups based on the unstimulated saliva flow rate [42]		18 subjects were taking medication knows to provoke xerostomia [42] An unstimulated saliva low rate is a great indicator for increased caries risk [42]

5 Traumatic and stressful events in the dental clinic		PTSD∗ manifestation [44]				

6 Bipolar disorder	Excessive tooth brushing and/or flossing	Affected mucosa or deficits at teeth cervical/gingival levels [14]				

7 Schizophrenia	Up to 50% reduced attendance to dental professionals >30% brushing frequency [13]	↑ Tendency to develop TMD∗ [41] Considerable throwback in the diagnostic process [41] ↓ Response to prolonged pain as opposed to acute [45] Misdiagnosis of TMD Higher prevalence of bruxism [45]	Clinical trial [45]	77 psychiatric patients under treatment with mostly dopamine antagonists + 50 healthy individuals as control [45] 15 schizophrenic patients with 1 never being admitted to the hospital or receiving neuroleptic treatment [46]	Psychiatric and/or schizophrenic patients + healthy controls	Lack of pain complaints suspected to be an ubiquitous dulling reply to pain connected with blunted replies that they present also to pleasure and basic emotions [41, 47] Tinnitus—being mistaken as possible auditory hallucinations [46] > Altered diagnosis of the patient's mental status [46] Almost 50% of the psychiatric group presented evident abnormal attrition in contrast with 20% in the control group along with significant differences for mean muscle and joint sensitivity to palpation and the range of mouth opening [45]
	Hypoalgesia					
	Auditory manifestations of the stomatognathic deficiency (such as ear fulness, hearing loss perception, and tinnitus)					

8 Psychiatric patients	Increased consumption of sugary and carbonated drinks [48, 49] Losing interest in performing hygiene activities, oral hygiene included [50, 51]	Creating a favorable environment for caries occurrence A possible accentuation of the symptoms of mental disorder through overconsumption of caffeinated soft drinks [49]	Cross-sectional population-based survey [48] Case report [49] Clinical trial [51]	7305 adolescents [48] 1 40 years old woman [28] 55 patients + 19 healthy individuals as control group [51]		Strong correlation between soft drinks consumption and mental distress [48] The increased consumption of sugary and carbonated drinks might be because of the altered taste perception [50, 51]

∗BMS: burning mouth syndrome; ∗TMJD: temporomandibular joint dysfunction; ∗DMFT: decayed, missing, or filled teeth; ∗DMFS: decayed, missing, or filled surfaces; ∗PTSD: posttraumatic stress disorder; ∗TMD; temporomandibular disorders.
